# Ritual plants of Muslim graveyards in northern Israel

**DOI:** 10.1186/1746-4269-2-38

**Published:** 2006-09-10

**Authors:** Amots Dafni, Efraim Lev, Sabine Beckmann, Christian Eichberger

**Affiliations:** 1 Institute of Evolution, University of Haifa, Haifa, 31905, Israel; 2 Department of Eretz Israel Studies, University of Haifa, Haifa, 31905, Israel; 3 Vigla Panagias, 72100 Agios Nikolaos, Crete, Greece; 4 Department of Organismic Biology, University of Salzburg, Hellbrunnerstr. 34, A-5020 Salzburg, Austria

## Abstract

This article surveys the botanical composition of 40 Muslim graveyards in northern Israel, accompanied by an ethnobotanical study of the folkloristic traditions of the use of these plants in cemeteries.

Three groups of plants were found to be repeated systematically and were also recognized for their ritual importance: aromatics herbs (especially *Salvia fruticosa *and *Rosmarinus officinalis*), white flowered plants (mainly *Narcissus tazetta, Urginea maritima*, *Iris *spp. and *Pancratium *spp.) and *Cupressus sempervirens *as the leading cemetery tree.

As endemic use we can indicate the essential role of *S. fruticosa *as the main plant used in all human rites of passage symbolizing the human life cycle. The rosemary is of European origin while the use of basil is of Indian influence.

The use of white flowers as cemeteries plants reflects an old European influence and almost the same species are used or their congeners. Most of the trees and shrubs that are planted in Muslim cemeteries in Israel have the same use in ancient as well in modern European cultures.

In conclusion, our findings on the occurrence of plants in graveyards reflect the geographic situation of Israel as a crossroads in the cultural arena between Asia and Europe. Most of the traditions are common to the whole Middle East showing high relatedness to the classical world as well as to the present-day Europe.

## Background

Plants were used to mark rites of passage both in human and in the annual cycle, as well as to decorate and to symbolize [[1]:262]. Plants, especially flowers, have been used at funerals in many cultures [[1]:66–70; [2]:165–167; [3]: 3–5]. Folkard [[4]:193] wrote, "All nations at different periods seem to have delighted to deck the graves of their departed relatives with garlands of flowers. The association of certain trees and plants with death and its glooming surrounding dates from a period remote and shadowy in its activity".

A century later Vickery [[5]:196] noted: "Flowers symbolize human mortality, and are equally symbolic of resurrection and rebirth, springtime and autumn, renewal and decay, and have long continued to provide consolation and hope at critical times in man's life".

In the New Kingdom of Ancient Egypt (16 – 12 centuries BC.) flowers of a particular sacred tree were considered life giving, so they were called "flowers of life". These flowers, and flowers of lotus, were used in funerals. Statues and coffins of the deceased were decorated with garlands of flowers [[6]:89].

According to Folkard [[4]:194] "The flowers strewed over graves by the Greeks were the Amaranth, Myrtle, and Polyanthous (=*Narcissus*)... The ancient Christians would choose different plants according to the age of the dead person: the flowers so used were deemed typical of the dead: to the young were assigned the blossoms of spring and summer, to middle age, aromatic herbs and the branches of primeval trees".

Roman funerals demanded a lot of flowers. The corpse was adorned as an expression of honour and affection. The funeral urn was similarly decorated. Flowers were scattered on the guests at the funeral banquet, and wreaths of dry and artificial flowers were placed at the tomb. After the funeral, fresh flowers, especially roses, lilies and violets, were used to deck out tombs as a memorial to show that the dead were still remembered [[1]:67; [4]:195; [7]:151].

The ancient Israelites differed from other religions: "The culture of flowers had put down deep roots in most societies of the Mediterranean and the Near East, being rejected only by ancient Israelites. As we have seen the Israelites accepted neither the sacrifices made to their neighbour's gods nor the garlands that accompanied these offerings" [[1]:70]. Therefore, as a rejection of their neighbours' "idolatry", they did not display flowers or plants at their ceremonies. In modern times Orthodox Jews do not use flowers in ceremonies, synagogues, cemeteries or even in houses [[1]:46–47]. Vickery [[5]:187] mentioned "In communities where burial takes place very soon after death, as is the case with Orthodox Jews, flowers are rarely associated with mourning, and the substitutes are mainly stones". While Goody [[1]:46–47] stated "Down today one finds stones not flowers in the cemetery, and then as reminder rather than offering". Secular Jews bring garlands of flowers and leave them on the tombs, but the ultra-religious are forbidden to plant any plant or flower upon graves [[8]: IIV; 34–35].

Interestingly, several authors [9-11] dealing with Muslim funeral customs, don't mentioned any ceremonial use of plants of any kind. While Spoer and Spoer [[12]:131] mentioned that "sweet smelling herbs" are placed on graves in Palestine.

The present article is a survey of the plants ritually used in Muslim graveyards of in northern Israel in the context of their cultural and ethnobotanical background.

## Methodology

The field study (1999–2005) was carried out in 35 Muslim villages in northern Israel, mainly in the Galilee. Field observations included recording plant species in the graveyards. Oral interviews were held with 80 informants, 48 who are cited personally (Appendix 1). The average age of the informants was 59.3 (SD = 18.1) years. Respondents were 41 males and seven females. In general women were reluctant to be interviewed, and when they agreed the interview was held in the presence of other family members.

The informants were mainly chosen according to their knowledge of common traditions and/or religious status. In each village we made a preliminary survey to locate people who are regarded as well immersed in local traditions and/or in religious customs.

The informants were asked about the ritual and religious importance of the plants found in the cemeteries, and why they were planted there. We used interview techniques to avoid a non spontaneous atmosphere and to overcome the reluctance of the informants to cooperate, Most informants refused to be videotaped or tape recorded. The botanical survey covered 40 cemeteries. We tried to avoid modern irrigated plots, on which many ornamental plants are grown. Cemeteries older than 50 years were considered "traditional". For each cemetery we listed the 20 most common plant species. Two parameters were calculated for each plant species: frequency (percentage of cemeteries in which the plant species appears) and dominance (percentage of cemeteries in which the species is the most common one).

## Results

We divided the list of recorded plants and the oral information gathered at the cemeteries into three categories based on the botanical characters of the plants: 1. Plants with aromatic leaves (Table 1); 2. White-flowered plants (Table 2) and 3. Trees and bushes (Table 3). These categories were found to correlate, more or less, with oral ethnobotanical evidence.

**Table 1:** Plants used in graveyards – aromatic plants.

**Species**	**Frequency of appearance and dominance (%)**	**References from the Middle East and adjacent countries**	**References from other regions (also for funerals, immortality and mourning symbols and death myths)**
Three-Lobed Sage – *Salvia fruticosa *Miller (=*S. triloba *L.f) (Lamiaceae)	93.1 (68.9*)		*S. officinalis* (Europe [20, I:666]; England; [14:102])
Rosemary – *Rosemarinus officinalis *L.** (Lamiaceae)	62.0 (10.3*)	Iraq and Iran (the Christian section of the Mandanean [17:183])	Ancient times [20; II:645–647]; Europe [2:168; 4:196], England [15:428,438; 56:138; 60:144; 61:150–151]; Cyprus [5:184,187,188]
Basil – *Ocimum basilicum *L. **^ (Lamiaceae)	65.5	Iran [51:191; 28:157]; Egypt [23:95]	Italy and Greece [20;II:54,]; India, (*O. sanctum*) [26:17–18; 62:22;])
Mint – *Mentha *spp.** (Lamiaceae)	50.0		Ancient Egypt [22:214–215; 63:120]; Ancient Minoan [64:6]; Ancient Greece [20;II:366; 65:121; 66:518; 67:XIX]
Myrtle – *Myrtus communis *L. (Myrtaceae)	37.9	Lybia [23:319]; Turkey [68:187; 69:62]; Syria [12:131]	Ancient Greece [ 4:194; 21:54; 58:14,82f; 65:49; 70:12,123ff; 71:8,94f, 426; 72: passim; 73:15,33; 74:tab 154; 75:18]; Rome [2:167; 4:465,476,487; 20;I:442; 21:54; 37:144; 76:49; 77:30]; Spain (Muslims,15^th ^century, [78:39]); England (16–17^th ^centuries, [14:102])
Rue – *Ruta chalepensis *L. (Rutaceae)	34.4		Spain [66:241]; England [5:184; 11:102]
Lemon verbena – *Lippia citroiodora *(Ort.) HBK ** (Verbenaceae)	31.0		
Rose – Scented Geranium – *Pelargonium graveolens *(L.) L'Her ex Ait. ** (Geraniaceae)	31.0 (7.5*)		
White-Leaved Savory – *Micromeria fruticosa *(L.) Druce (Lamiaceae)	27.5		
Lavender – *Lavandula *spp.** (Lamiaceae)	20.6		
Tagetes – *Tagetes sp*. **^ (Asteraceae)	20.6		
Wormwood – *Artemisia arborescens *L.* (Asteraceae)	17.2		England [5:184–185]
Jasmine – *Jasminum *sp.** (Oleaceae)	17.2	Lybia [4:191]	Spain (Muslims,15th century, [78:39])
*Thyme – leaved Savory Satureja thymbra *L. (Lamiaceae)	6.8		

* = As a dominant plant

** = Non-indigenous species

^ = Annual species.

**Table 2:** Plants used in graveyards – white-flowered plants.

**Species**	**Frequency of appearance (%)**	**Reference from the Middle East and adjacent countries**	**References from other regions (also for funerals, immortality and mourning symbols, and death myths).**
Narcissus – *Narcissus tazetta *L. (Amarylliadaceae)	41.3		Ancient Greece [21:68–269; 35:248; 36:430; 37:148]; Rome [77:31]; England [30:148]
Sea squill – *Urginea maritima *(l.) Baker (Liliaceae)	37.9		
Day Cestrum – *Cestrum diurnum *L. (Solanaceae)	27.5		
Iris-Mainly *Iris *albicans W. Barley and *I. mesopotamica *Dyes (Iridaceae)	24.1	Palestine [39:296; 81:812]; Syria (16th century, [82:63]; North Africa [S. Jury Pers. Comm.]	Muslim cemeteries from Spain to Kashmir [21:64–65]. India [Muslims, 78:39]
Sea daffodil – *Pancratium maritimum *L. and *P. parviflorum *Delile (Amaryllidaceae)	22.2		Late Minoan [7:176–184]
Rose – *Rosa *sp. (Rosaceae)	20.0		Ancient Greece and Rome (Red flowers! [83:219] and references therein); England [5:184–185; 14:102]
White Arum-Lily – *Zantedeshcia aetiopica *Sprengl. (Araceae)	20.0		France [1:284]
Asphodel – *Asphodelus ramosus *Miller (=*A. aestivus *Brot.) (Liliaceae)	6.8	Turkey [75:18]	Minoan Crete [84:55]; Ancient Greece, (symbol of death, [21:65; 38: XI, 539–543, XXIV, 13;], planting on graves [31:157; 75:18; 85:62], as a funeral plant, [86:300]). Modern Greece [87:100–101].
Virgin Mary Lily – *Lilium candidum *L. (Liliaceae)	0		Rome [77:30]; Europe [20, II:324]; Germany [31:12]; England [14:102; 30:228]

**Table 3:** Plants used in graveyards – trees and shrubs.

**Species**	**Frequency of appearance and dominance (%)**	**References from the Middle East and adjacent countries.**	**References from other regions (also for funerals, immortality and mourning symbols and, death myths)**
Cemetery Cypress – *Cupressus sempervirens *L. (Cupreessaceae)	68.9 (20.6*)	Iran [86:18; 88:132; 89:30]; Turkey [42:182; 69:63; 91:350; 92;II:21; 93:219; 94:115; 95:136,146]; Lebanon [96:205]; Egypt [97:431]	Ancient Greece, [2:80; 17:34; 42:182; 43:298; 57; X:106–142; 98;11.4; 99: 8,24,7; 100:27; 101:111.4, 120.75; 102:654; 103:264,267; 104:32; 105:157]; Europe in general [31:36; 54: 190–191; 55:XVI,60; 56:IV,507; 47:39]; China [2:28; 106:124]
Olive – *Olea europaea *L. (Oleaceae)	41.3		Ancient Greece [3:308; 100:27; 107:4.84] and also Rome [56: 230; 20, II:500; 89:373]
Date palm – *Phoenix dactylifera L*. (Palmae)	37.5	Egypt [109;II:132; 110:464; 111:431]	Europe [2:9; 20;I:129; 45:191]
Ficus – *Ficus *spp. (Moraceae)	22.5		
Butcher's Broom – *Ruscus aculeatus *L. (Dioscoreaceae)	20.0		
Oleander – *Nerium oleander *L. (Apocynaceae)	20.0		Rome [77:30]
Carob – *Ceratonia siliqua *L. (Caesalpiniaceae)	20.0		
Christ's Thorn Jujube – *Ziziphus spina-christi *(L.) Desf. (Rhamnaceae)	10.0	Middle East [50:passim]	
Fig – *Ficus carica *L. (Moraceae)	10.0		England (19^th ^century [14:102])
Laurel – *Laurus nobilis L*. (Lauraceae)	2.5		Ancient Greece [3:107–108; 45:191]; Rome [77:30]; Europe in general [20, I:174; 108:174; 112:174]; England [14:102; 15:438; 30:7];

* = As a dominant plant

### Aromatic plants

Data concerning the presence of aromatic plants in the surveyed cemeteries are presented in Table 1. The following points emerge:

1. On average, 6.0 (SD = 4.3; range 0–14) aromatic species were present in each cemetery.

2. Half of the recorded species were members of the family Lamiaceae.

3. Only one cemetery had no aromatic plant.

4. The most frequent as well as dominant species in cemeteries was *Salvia fruticosa *and the second was *Rosmarinus officinalis*.

5. In 18 cemeteries wreaths of aromatic plants were placed on the fresh graves at the funeral and/or during later visits (especially *Salvia *and *Ocimum*).

6. The presence of wild aromatic plants in the nearby natural habitats did not correlate with species diversity of such species on graves. In fact, 66% of the aromatic species in cemeteries were not indigenous.

7. In the traditional cemeteries all the plants were perennial, only recently the adoption of irrigation systems allows maintenance of annuals such as basil (*Ocimum*).

8. In the old traditional cemeteries (mostly abandoned or neglected today) *S. fruticosa *and *R. officinalis *were almost the only aromatic species present.

In attempt to uncover the possible role of *Salvia fruticosa*'s use in funerals and cemeteries informants were asked "Why is *S. fruticosa *used in funerals and planted at graves?". We received the following answers (Bold number in parenthesis indicates the informant identity, see Appendix 1):

1. "The angels like a good odour and come to the fragrant plants, and they also transfer the prayers to the dead" (**7**). "We place *Salvia *in cemeteries because it gives a good odour. When a person is deceased the angels come to visit him and they like good odour – that is why we put *Salvia *there" (**7, 8, 16, 19**). This note is related to the common belief that angels go to judge the dead person in his grave [[10]:74; [11] passim; [12]:130,134].

2. "Fragrant plants are planted in cemeteries to counteract the unpleasant smell; we have to recall because the burial was superficial" (**15**). "For a good odour in the cemetery" (**17, 28, 33, 36, 38, 41, 43, 49**).

3. "We put *Salvia *on graves because it gives medicine, "*barakeh" *(a divine blessing) and good odour" (**11, 12, 13, 14, 18, 20, 21, 24, 29**). "Because it is a blessed and sacred plant that gives good odour and it is healing" (**15, 21, 25, 26, 34, 43, 44**).

4. "Because it is evergreen and has a good odour" (**26, 30, 40**).

5. "As a token of honour and appreciation for the deceased" (**9, 24, 39**).

6. "Because of its good odour, beauty, and medicinal power "(**20, 31, 35, 48**).

7. "It has fragrance; it is a beloved and respected plant. It accompanies man during all stages of his life" (**15, 18, 32, 33**).

8. "We purify the dead with *Salvia *and for good odour" (**10, 38**).

9. "It expels the Satan and the evil eyes" (**7, 19, 23, 33, 45, 47, 48**).

10. "There is justice in this plant" (**34**).

11. "For the blessing of the dead" (**48**).

Three-Lobed Sage (*Salvia fruticosa*) has an important role in daily rituals in the Muslim life-cycle in Israel and today it accompanies each person as a main ritual plant from birth to death:

1. When an infant is born he is placed on a bed of fresh leaves of three-lobed sage, and the mother drinks three-lobed sage tea (**1, 7, 15, 24, 34, 37**). When infant is born a ceremony called *mauled *is performed. A sheep is slaughtered and all the friends and the relatives are invited. The Sheikh arrives and reads chapters from the Quran. A substantial number of three-lobed sage leaves are mixed with barley and placed on a tray near the Sheikh. The Sheikh reads the (appropriate) chapter and each of the guests takes a fistful of the mixture in a small packet. At home this mixture is used to prepare an incense against evil eye and demons; it is placed on burning coals and then the house is blessed with a good fragrance and the demons are expelled (**1,12, 27**).

2. At every wedding, and any other family feast, incense of three-lobed sage leaves is placed on burning coals. It is used against evil eye and to expel demons (**1, 2, 18, 27**).

3. Garlands of three-lobed sage for incense are left at the graves of saintly people as well as in front of sacred trees, for the private use of the visitors who pray and burn incense in honour of the holy man (A. Dafni personal observations).

4. Garlands of three-lobed sage are used in funerals and also placed at graves; the dead body is placed on a layer of fresh three-lobed sage leaves (**2, 24, 34, 42**).

One informant (**15**) summarized the importance of three-lobed sage in the daily local ritual: "By placing three-lobed sage on the graves, a connection is sustained from birth to death", while another person (**27**) said "The three-lobed sage accompanies man in all stages of his life".

### White-flowered plants

Data concerning the presence of white flowered flowers species in the surveyed cemeteries are presented in Table 2. We report the the following trends:

1. White flowers are more common in old traditional cemeteries, some of which are abandoned or neglected today.

2. The commonest white-flowered plants in traditional graveyards are *Iris *spp.

3. Today the most common plants in new cemeteries are *Narcissus tazetta *and *Urginea maritima*.

4. White flowered species are usually taken from the local nearby vegetation. *Pancratium maritimum *may be found in cemeteries along the seashore, which is their original habitat, while *Pancratium parviflorum *is evident in cemeteries near calcareous habitats, in which it grows naturally.

5. *Narcissus tazetta *(and other white cultivars) and *Iris *spp. are scattered without any clear geographic or ecological pattern.

6. *Asphodelus ramosus *is quite rare as a graveyard plant.

7. All the species in this group are perennial geophytes, which need no cultivation.

When we asked the informants "Why are white flowers planted on graves?" we received the following answers:

1. "White flowers are signs of "something good" (**26**).

2. "White is the way to Paradise" (**28**).

3. "White flowers are pure like the soul of the deceased" (**4**). "The white colour a sign that a man is pure and clean" (**10**).

4. "The white flower recalls the colour that characterizes the Haj, white colour is beloved by God, and white colour will erase the deceased's sins" (**7**).

5. "Because of the beauty, and to honour the deceased.... white colour .... A bride wears white, every dead person is wrapped in white cloth, the pilgrims to Mecca wear identical white clothes to show that all are equal" (**8**).

6. "White colour is the colour of birth (the baby is wrapped in a white cloth), of a wedding (the bride's dress), and of death (the white shroud)...life commenced with white and ended with white (**27**).

7. "The white flower recalls the colour of the pilgrim's garment ...white is preferred by God and will redeem the dead person" (**34**).

8. In this connection we heard the following story "A king had a lovely daughter. One day the daughter was violated, and a girl who disguised herself a boy was accused of the rape. When the head of the accused girl was cut off, her real identity was disclosed and the people regretted their deed. *Zambak *(a general name for white flowers like *Lilium, Iris *and *Pancratium*) flowers were planted on the daughter's grave. Because the raped daughter had acquiesced to the violation she was buried and a carob tree (which is regarded as a 'bad tree') was planted over her grave" (**5**).

### Trees and shrubs

Data concerning the presence of trees and bushes in the surveyed cemeteries are presented in Table 3. From our observations and Table 3 we can conclude:

1. Cypress is the most frequent (68.9%) as well as the only dominant plant (20.6%). It is planted especially as a fence round cemeteries and also between graves. In old traditional cemeteries, cypress is almost the only tree.

2. Palms and olives are planted among graves and branches of both species are frequently placed on fresh graves. An interesting feature is the abundance of palm branches placed on tombs on '*Id el-fitr *(the feast at the end of Ramadan).

3. *Ziziphus spina christi *is not planted but is a component of the natural vegetation.

4. Several species of cultivated fruit trees are planted today in cemeteries (*e.g*. pomegranate, oranges, almonds, mulberry, and loquat); at present it is hard to see any pattern in this trend.

5. In one Bedouin village (Arab al Aramshe, **40–43**), branches of *Laurus nobilis *are placed on graves.

6. In one Village (Akhbara), young shoots of *Myrtus communis *are placed on fresh graves (**46–48**, and our personal observations). In this village myrtle is grown commercially for the Jewish religious market for use at the Tabernacle Festival.

The following answers were given, by the informants, to our question, "Why are trees planted in graveyards?"

1. "Shrubs and trees are planted on graveyards to create a connection between the deceased and his God" (**21**).

2. "Each tree protects the grave because it is green – a protection against evil events" (**18**).

3. "Every planted tree on a grave is praising the merciful god, on behalf of the deceased, all the time" (**27**).

4. "Green trees reduced the punishments inside the grave" (**39**).

## Discussion and conclusion

### Aromatic plants

The custom to put aromatic plants in cemeteries is deep-rooted in human history (Table 1). One may point out three reasons for the use of aromatic plants in cemeteries as well as in funerals.

1: Seaton [[13]:11] explained the use of aromatic as follows" Flowers, especially aromatic ones, played a hygienic role in handling of the dead in those days before embalming. While according to others: "Aromatic herbs and strongly scented flowers may suggest the original use of funeral flowers... to mask the odour of decaying flesh" [[5]:186–187]. " Aromatic herbs and strongly scented flowers were invariably used to hide the odour of decay in the days when the dead were laid out, often several days, in private houses before burial" [[14]:101] see also [[4]:189] concerning Wales, [[15]:428] for England and [[16]:47] for ancient Greece, Rome and Arabia). In Iraq and Iran the Christian sect of the Mandaeans used to smell sweet smelling flowers "so that the smell of death shall not reach them" [[17]:183]. The Armenians in Constantinople would plant Turpentine Terebinth (*Pistacia terebinthus *L.) on graves and use the aromatic resins to mask the smell from the graves [[18]:67]. It is noteworthy that in Rome perfumes were used in funerals for the same purpose [[19]:286]. Our findings corroborate this general trend.

2. De Cleene and Lejeune [[20]: I: 647] explain the use of rosemary in funerals: "It was in fact...that its aroma conserved the dead body and that its evergreen leaves guaranteed immortality. Since incense was expensive, there was a preference for the aromatic Rosemary in religious ceremonies in antiquity, especially burial" (see also [[21]: 89; [22]:315]).

3. The main aromatic plants have a special ritual importance throughout the human rites of passage, from birth through weddings to the grave.

To summarize the informants' views on *S. fruticosa *one may recognize the respect devoted to these specific plant as an important medicinal, as well as a ritual plant that accompanied man from birth to death. The informants indicated the special reverence to *S. fruticosa *as a blessed plant beyond its physical qualities. There were some nuances and different views among the informants, but all expressed the high importance of this plant in daily life. Hardly any cemetery is devoid of the prevalence of this species (Table 1).

Rosemary has been very common in Europe as a funeral plant since ancient times [[20], II: 645–647; [22]:315–316] and was used at weddings as well [[5]:364; [20], I:647–648], the explanation given is "It may seem surprising to learn that the plant's symbolism is linked to birth, sexual capacity and death". The reason for this is probably to be found in the belief that this aromatic plant keeps at bay evil spirits, which might otherwise exercise their harmful influence at life's important rites of passage (birth, marriage, and death) [[23]:191–192].

Several species of basil, especially "tulsi" (*Ocimum sanctum *L.), are regarded as the most sacred plants in the Hindu religion and therefore Basil is found in almost every Hindu house throughout India. The leaves are used for different ceremonies as: births, and weddings, sacred rituals, as well as in funerals [[24]:44–45; [25]:34–35; [26]:38–39].

Zohary and Grebel [[27]:613–614] noted the ritual role of the myrtle as a funeral as well as a wedding plant in Europe. In Judaism it is well known as a wedding plant (Jerusalem Talmud, *Peah*, 1: 15, and 4). In our survey we found that myrtle is commonly planted in graveyards (Table 1.) but only in one village young shoots are placed directly on the graves.

### White flowered plants

White flowers are used in cemeteries and funeral due to the following reasons:

1. The white colour is a symbol of purity. According to Puckle [[2]:169] the fact that white flowers are almost exclusively used at funerals reminds us that they are a special token of purity. Our informants stressed the role of white colour as a symbol of purity and the rites of passage in human life as a reason why white flowers are planted on graves. Crowfoot and Baldensperger [[28]:126] recorded a story that clarifies this trend. An innocent girl from the village of Artas (in the Judean Mountains) was killed by her brother, who claimed that she had brought the family fame into disrepute. A white flower, "as it were the flower of gourd plant climbing upwards", grew on her grave and all the people of the village and the area knew and said it is because she was innocent. We found a similar story, which reaffirms the popular belief that white flowers are emblem of the purity of the deceased. White flowers were frequently used for funerals and as graveyard plants in Europe [[29]:157; [30]:220–221, 228, 253]. In German folklore the soul was supposed to take the form of a flower, such as a lily or a white rose. White lilies are said to spring from the grave of one unjustly executed as a token of the person's innocence [[31]:12]. In Britain white flowers are "funeral flowers" and must not be brought indoor [[32]:63].

Although the Madonna Lily (*Lilium candidum *L.) was not found in our survey it is worth mentioning in this context. The lily was popular among the Greeks and Romans, and was dedicated by the Christian Church to the Madonna, probably because its delicate whiteness was considered a symbol of purity [[33]:482].

According to De Cleene and Lejeune [[20], II: 324] the lily became a flower of death, graves, and the churchyard in Europe. It is preferably planted on the graves of children and virgins. As a grave flower it also symbolizes love, which transcends death. According to our survey the lily is apparently a "Christian plant" and not used by the Muslims for any ritual uses. As a wild plant it is very rare locally, although the cultivated species is quite common in Christian monasteries. Lily images can be found in Christian cemeteries engraved on gravestones (*e.g*. the Templar Cemeteries in Haifa as well as in Jerusalem: A. Dafni (Personal observations 20.4.2004 and 13.6.2004 respectively), as in Christian graves in the States [[34]:283–284].

2. Old myths related to the underworld – In the Greek Mythology narcissus is related to the underworld. Gaia, to please Hades, made the narcissus (*Narcissus poeticus *L.) grow so that its sweet perfume would entice Persephone, the daughter of Demeter into the underworld. This myth, in association with the scent of the narcissus, goes back the custom that has persisted to modern times of bedecking the dead and their graves with these flowers [[21]:68–69; [35]:248]. When the dead went into the presence of the gods of the underworld, they carried crowns of narcissus that those who mourned had placed in their white hands when the last good-byes were said [[37]:148].

Asphodel was mentioned in Homer [[38]; xi, 539, 543; xxiv, 13] as growing in the meadows of the land of departed [[21]:65]. The Greeks also planted asphodel round their graves, as the fleshy roots of these plants were supposed to nourish the dead [[1]:191; [31]:157].

Iris accompanied the souls of mortals to their rest along a path made by the rainbow, whose iridescent colours the iris possesses [[21]:64–65].

The Sea Squill is very common especially in old cemeteries as a connection to God as "messengers of the praying to the dead" (owing to its very deep roots). One of the local names *basl el maytin *(bulb of the dead) reflects this popular belief [[39]:295–296; [40]:87], it is also called "*basl el makbara*" (the cemetery's bulb, **18, 25**).

3. White is the Muslim colour of mourning – This explains the occurrence of irises from Spain to Kashmir [[21]:64–65] as well as in North Africa (S. Jury, University of Reading, Pers. Comm. 18.10.05)., as we already found.

4. Symbol of repentance – In Britain roses (usually white), rue, hyssop, thyme, or wormwood, all considered symbolic of repentance, and were arranged on coffins [[5]:184–185].

### Trees and shrubs

Some trees and shrubs are planted in graveyards due the following reasons:

1. Evergreen plants – These shrubs and trees are symbol of eternity, according to Puckle [[2]:167], (see also [[42]:182] for cypress). The ongoing custom of planting certain kinds of trees in graveyards is due to their appearance, which by association promotes their use as symbols of immutable grief. Myrtle was regarded to have "sombre appearance" [[2]:99] and cypress as "dark and gloomy...to express sorrow [[58]:18]. Cypress was planted in cemeteries also because its dark green branches [[43]:154; [52]:194]

Evergreen trees were carried at funerals as a symbol of the soul's immortality. Jenner [[43]:154] mentioned "...the cypresses. From its not losing its leaves in wind it has adopted as the image of the just man who preserved his virtue.... In this significance it was placed on gravestones...from its dark and sombre colouring it has been used as a symbol of mourning and death, and it is almost universally used in cemeteries ".

In England until the 19^th ^century mourners customarily followed the corpse to the grave carrying rosemary, ivy, laurel, and other evergreens as a sign of immortality [[14]:102]. According to Maurin [[44]:196] evergreen foliage in cemeteries is held in contrast to the decay of the body. Folkard [[4]:189] noted "the belief in a future existence doubtless led to the custom of planting trees on tombs, especially the cypress, which was regarded as typical both of life and death. The tree growing over the grave, one can easily imagine, was looked upon by the ancients as an emblem of the soul departed become immortal".

The Greeks considered the myrtle a symbol of love and immortality, due to its evergreen character [[2]:167; [20]: 2003 I: 442; [37]:144].

2. Triumphal species – Laurel and date palm were use in Europe at funerals to "symbolize victory over adversity in life" [[45]:191], (see also [[2]:99; [20]: I; 129; [46]: 375]). In many cultures the date palm is a symbol of military victory and eternal fame. In funeral wreaths the palm is a mark of honour for the deceased. This wreath also symbolizes triumph over death [[2]:168; [20]: II: 213]. In later Greek mythology date palm is closely associated with Apollo and his twin sister Artemis as well as Athena. Therefore the date palm often appears as a symbol of light and victory [[35]: 48ff]. Cypress is frequently represented on ancient gravestones [[47]:13]. The unusual combination of cypress branches with palm fronds represents the triumph over death [[20]: II: 401]. Today, on Mount Kackar (North-eastern Turkey), there are depictions of the cypress tree on tombstones of men (A. Dafni personal observations).

3. Remnants of tree worship – Puckle [[2]:165] related trees in cemeteries to tree worship. Planting certain trees in the churchyard and cemetery is an almost a universal custom. In Israel tree worship is still widespread, and many of the trees are associated with graveyards and are especially venerated (**50, 51, 52**, [[48-50], passim]).

4. Trees are mentioned in religious scripture – *Ziziphus spina christi *(L.) Desf. has a special role as a cemetery tree due to its being mentioned in the Quran (LIII: 13–18; LVI: 28–32) under the name of "lote tree" [[51]:65–74] and, accordingly, this species is highly respected by the Muslims through the Middle East [[50]: passim]. In several countries [[50] and references therein] the bodies of dead Muslims were washed with water in which the leaves of *Ziziphus spina – christi *were soaked. That is the reason why this species is kept in cemeteries.

The olive is highly praised locally as a "blessed tree" (*al-shajart al-mubraka*) and is mentioned several times in the Quran [[51]:34–39], in North Africa people say that the olive "looks towards heaven to praise God" and a cut, of even unproductive twigs hearted their deep religious feelings [[52]:512–513].

The date palm has notable religious importance in Islam [[51]:22–27] everyone who has performed the Haj pilgrimage decorates the entrance to his house with an "arch" of palm fronds. Anyone entering the house has to pass under these branches. Palm branches are carried in front of every Muslim funeral procession (in Palestine) and later laid over the grave. Four such branches are put into the ground, one at each corner of the tomb, in such a way that their tops touch each other. Often these branches are interwoven with flowers. It is a common belief that as long as they remain green the deceased is able to praise God. In this way God partly, or wholly, forgives the deceased for his misdeeds in life. Many tombstones have small palm branches engraved on them [[53]:153]. Graves are copiously adorned with palm fronds by visitors to cemeteries during festivals such as *Id El Fitr *at the end of the Ramadan (A. Dafni Acre, Oct. 2004, personal observation).

5. The trees do not regenerate after cutting – Scholars have explained the mourning symbolism of cypress by the fact that it does not send up new shoots after having been cut [[54]:192].

6. Relation to the underworld – The cypress, called by Greeks and Romans alike "the mournful tree", was also sacred to the rulers of the underworld, and to their associates, the Fates and the Furies. Accordingly it was customary to plant it by the grave, and, in the event of a death, to place it either before the house or in the vestibule, to warn those about to perform a sacred rite against entering a place polluted by a dead body [[47]:39;55:XVI, 60; [56]: IV, 507;]. According to Ovid [[57], X: 106–142], this tree was named after Kyparissos, Apollo's favourite. This young boy accidentally slew his beloved stag. The boy was struck with such anguish that he besought the gods to doom his life to everlasting gloom. They, in compliance, transformed him into a cypress tree.

7. Mythical powers – In Antiquity the laurel was seen as physically and morally purifying [[45]: 288–290,293,302]. Laurel was used in funeral rites as an attempt to preserve the continued vitality and strength of the Roman people because it is an emblem of prosperity and agricultural productivity [[3]:107–108].

In conclusion, our findings on the occurrence of plants in graveyards reflect the geographic situation of Israel as a crossroads in the cultural arena between Asia and Europe. Most of the traditions are common to the whole Middle East showing high relatedness to the Greco-Roman as well as to the present-day Europe.

As endemic tradition we can indicate the essential role of *S. fruticosa *as the main plant used at all human rites of passage symbolizing the human life cycle. Rosemary is of European origin while the use of basil is of Indian influence.

The use of white flowers as cemetery plants may reflect an old European influence and almost the same species are used or their congeners.

The cypress is the main Muslim cemetery' tree as in ancient as well as modern European cultures. The date palm and the olive, the next most prevalent trees in cemeteries, reflect the status of these two species in the Quran as blessed trees.

## Appendix

List of the cited informants.

The number after the name indicates the age of the informant followed by the name of village and the date of the interview.

1. *Shukri Araf*, 62, Maeelia, 26.6.00.

2. *Hamud Jaudat*, 30, Kabul, 18.8.00.

3. *Zaher Mahra*, *40*, Acre, 20.8.00,

4. *Amana Halifi, 63, Iblin, 1.12.00.

5. *Sheikh Hussien Shahin*, 70, Beit Jan, 12.9.00.

6. * Amana Izat Abu el Heija, 73, Kaukab Abu el Heija, 03.07.03,

7. *Raja Taha*, 54, Deir Hana, 31.12.03.

8. *Abbas Mustafa Daud*, 82, Dabburieyh, 7.1.04.

9. *Jafar Muhammed Sbeikhi*, 58, Shibli, 22.1.04

10. *Adel Abu Hamid, 57*, Kafr Manda, 3.6.04

11. *Jafar Muhammed Sbeikhi*, Shibli, 67, 22.1. 04.

12. *Araf Farooki*, 65, Tamra, 2.8.04.

13. *Ali El Anan-Abu Khaled*, 67, Salame, 2.8.04

14. *Makhmud Awad*, 68, Tamra, 2.8.04.

15. *Abbas Mustafa Daud*, 82, Tamra, 2.8.04.

16. *Raja El-Hatib*, 62, Dier Hana, 02.08.04

17. *Tamir Fallakh Mazarib*, 35, Mazarib (Beit Zarzir), 11.8.04.

18. *Abu Ma'aruf*, 75, Bueina-Nujeidat, 16.8.04.

19. *Abu Razze*, 95, Bueina-Nujeidat, 16.8.04.

20. *Hadi Samiyeh, 90, Mazra'a, 24.8.04

21. *Sheikh Akhmed-Abu Ammar*, 54, Majdal Kurum 28.8.04.

22. *Jamil Abu Raeed Arafat*, 71, Mesh'hed, 23.9.04.

23. *Abu Khaled Ali El Anan*, 67, Salame, 2.9.04

24. *Jamil Shibli*, Shibli, 34, 1.11.04.

25. *Makhmud Kmeidi *Abu Ikhia, 57, Shibli, 1.11.04.

26. *Makhmud Maslaha-Abu Karem*, 57, Daburrieyh, 11.11.04

27. *Mukhamed Taher*, 65, Rumana, 23.11.04

28. *Mukhamed Ali Fukra*, 58, Bueina-Nujeidat, 23.11.04

29. *Yusuf Nimmr Massar-Abu Lutuf*, 67, Sakhnin, 1.1.05,

30. *Muhana Dalashi, 61, Bueina, 1.1.05*

31. *Nasser Khalil*, 68, Sakhnin, 1.1.05

32. *Abdaala Ahab*, 60, Shibli, 3.1.05.

33. *Salman Shibli*, 46, Shibli, 3.1.05

34. *Na'amaeh Mukhamad Iktilat, 40, Daburieh, 3.1.05.

35. *Ahmed Abu Rakhman Iktilat, 82, Daburieh*, 3.1.05

36. *Sami Salman Shibli*, 31, Daburieh, 1.3.05.

37. *Abed Zuabi*, 61, Taibe, 28.1.05

38. *Ali Zuabi*, 30, Taibe, 28.1.05)

39. *Mustafa Daud*, 56, *Shibli*, 12.3.05

40. *Abdalla Taha*, 62, Shibli 12.3.05

41. *Fathi Ali*,, 55, Arab el Aramshe, 22.3.05

42. *Wakhid Ali*, 32, Arab el Aramshe, 22.3.05

43. *Wahib Ali*, 20, Arab el Aramshe, 22.3.05

44. *Rukkiah Maghis, 50, Jordikh, 22.3.05

45. *Umm Ommar, 75, Jordikh, 22, 3.05

46. *Akhmed Khaled Khleikhel*, 63, Akbara, 28.3.05

47. *Zahara um Mukhmed Khleikhel, 80, Akbara, 28.3.05

48. *Sa'eed Makhmud Khsein Khleikhel*, 80, Akbara, 28.3.05

* = Female
